# Heats of Mixing Using an Isothermal Titration Calorimeter: Associated Thermal Effects

**DOI:** 10.3390/ijms10072911

**Published:** 2009-06-29

**Authors:** Manuel Rodríguez de Rivera, Fabiola Socorro, José S. Matos

**Affiliations:** Departamento de Física, Universidad de Las Palmas de Gran Canaria, E-35017 Las Palmas de Gran Canaria, Spain; E-Mails: fsocorro@dfis.ulpgc.es (F.S.); jsmatos@dfis.ulpgc.es (J.S.M.)

**Keywords:** accuracy, excess enthalpies, isothermal titration calorimeter, liquid mixtures

## Abstract

The correct determination of the energy generated or absorbed in the sample cell of an Isothermal Titration Calorimeter (ITC) requires a thorough analysis of the calorimetric signal. This means the identification and quantification of any thermal effect inherent to the working method. In this work, it is carried out a review on several thermal effects, studied by us in previous work, and which appear when an ITC is used for measuring the heats of mixing of liquids in a continuous mode. These effects are due to: (i) the difference between the temperature of the injected liquid and the temperature of the mixture during the mixing process, (ii) the increase of the liquid volume located in the mixing cell and (iii) the stirring velocity. Besides, methods for the identification and quantification of the mentioned effects are suggested.

## 1. Introduction

The most used instruments nowadays to determine the excess molar enthalpies for liquid mixtures are the Flow Microcalorimeter (FM) and the Isothermal Titration Calorimeter (ITC). Both instruments are isothermal heat conduction calorimeters but their operation principles are different. In a FM, the mixture is produced when two liquids are injected simultaneously in the so-called “reaction zone”. When the stationary state is reached, it is supposed that the mixture is homogeneous and the heat of mixing is proportional to the stationary value of the calorimetric signal [1–2]. A drawback that this instrument shows is that an increase in the injection velocities may lead to the fact that the mixture area is located outside the detection area of the thermal flow. In order to avoid the situation described before, it is appropriate to use very low injection velocities that, at the same time, make clear a reduction of the calorimetric signal and of the relationship signal-noise [3]. However, this instrument is very easy to use as it allows obtaining the heat of mixing with very few operations, although it is necessary to operate within the margins established in the calibration [4]. In addition, the FM can be used with volatile liquids, even gases [5,6] and it can operate over extended temperature and pressure ranges [7].

The ITC also present a wide spectrum of use. Different magnitudes (enthalpies of solution, enthalpies of dilution, enthalpies of reaction in solution, enthalpies of mixing) can be measured with these instruments. When an ITC is used to determine heats of mixing of liquids, two operation modes are possible: (1) in the standard mode, a known amount of a liquid is injected on another known amount of a different liquid. The mixing process results in a calorimetric signal (the result of integrating the calorimetric signal, divided by the sensitivity of the instrument, provides the value of heat of mixing). (2) A second mode is the continuous-injection mode (at very low velocity) and, from a sophisticated calorimetric signal processing, the heat power that is developed in the mixing process is obtained as a function of the mole fractions [8,9].

In this study, a TAM2277-201/2250 calorimeter, by Thermometric, operating in continuous-injection mode has been used to show several thermal effects associated to: (i) the difference between the temperature of the injected liquid and the temperature of the mixture during the mixing process, (ii) the increase of the liquid volume located in the mixing cell and (iii) the stirring velocity. Although the role of the stirring speed is important [10], the impact of the first two effects is small when the excess enthalpies are large, but it is very significant when the magnitude of the enthalpies is relatively small. Furthermore, an operation model of the calorimeter, which quantifies the thermal effects mentioned above, is proposed. The model allows make corrections in the calculation of heat of mixing from calorimetric signal in which all the thermal effects overlap.

## 2. Experimental Section

The instrument used in this study was an ITC TAM2277-204/2250, by Thermometric. The calorimetric cylinder is submerged in a thermostatic bath TAM2277 that keeps constant the temperature with a precision of ±0.001 K, in stationary state. All the experiments shown in this work have been made at *T* = 298.15 K. The calorimetric cylinder contains the detection system in which it is inserted the mixture cell that has a useful capacity of 2.5 mL. The calorimetric signal is directly read by a digital multimeter HP3457A (10 nV resolution). The Joule calibration is carried out through a resistance of 50 Ω placed in the base of a cell and the feeding is done through a programmable DC power supply HP6633A. The dissipated power is determined at every time by measuring the voltage in the terminals of a standard 10 Ω resistor located in series with the Joule calibration resistor. This voltage is measured with a multimeter HP3478A (100 nV resolution). The injection system allows to inject 0.0832 μL per step of the motor, which, through a micrometric screw moves a billet that pushes the piston of a 50 mL Hamilton syringe. By programming the number of steps of the motor at every sampling period (Δ*t=1 s*), we obtain the injection flow. The injection flows used varied from 0.5 and 2 μL/s. In order to assure that the injected liquid acquires the temperature of the thermostat it has been disposed a coil of capacity 1.5 mL, previous to the mixture cell. The stirring system assures a homogeneous mixture at every time and its stirring velocity is programmed in every measure (0–240 rpm) through a programmable DC power supply Agilent E3640A (0–24V). All the described elements are controlled through the GPIB bus by a PC and the readings are stored for the subsequent analysis.

With regards to the calibration of the instrument, it is important to indicate that as electrical calibration is used to check the adequate operation of the calorimeter and to study the different effects that take place within it, the energetic dissipation in an electrical resistor is not equal to the energetic exchange in a mixing process. The electrical dissipation always happens in the same place, i.e., it is localized. However, the energy in a mixing process spreads all over the liquid domain. In short, the electrical and chemical calibrations lead to slightly different values for the sensitivity. This is the reason why it is necessary to carry out a chemical calibration, as described in a previous work [11]. Two binary systems (ethanol + water [12] and cyclohexane + benzene [13]) were used for the chemical calibration. Both systems have been proposed [12,14] as reference systems for testing mixing calorimeters. Finally, an average value (K = 391 ± 9 mV/W) was obtained from different working conditions. A comparison between our experimental results and those recommended in the literature, has been reported elsewhere [11].

## 3. Results and Discussion

### 3.1. Calorimetric Model

In order to obtain reliable values of heats of mixing, it is necessary to quantify the thermal effects mentioned in the previous section and to make the adequate corrections afterwards. With this purpose in view, the localized-constants model was used, which have been utilized acceptably to represent the operation mode of several conduction calorimeters [15–18]. The model considers the experimental device consisting of *N* domains each with a heat capacity *C_i_* and infinite thermal conductivity (being *T_i_* the temperature throughout *i-th* domain). Each domain is connected to the neighbouring domains through thermal couplings with thermal conductivity *P_ik_*. The power *W_i_* generated or absorbed in the domain of capacity *C_i_* is the sum of the stored power *C_i_* (*dT_i_*/*dt*) plus those transmitted by conduction to the thermostat (*T_i_*) and to the neighbouring domains:

(1) $$W_{i}=C_{i}\frac{dT_{i}}{dt}+\sum_{1}^{N}P_{ik}\left(T_{i}-T_{k}\right)+P_{i}(T_{i}-T_{0})$$

Moreover, to obtain heats of mixing of the liquid mixtures using an ITC, it is necessary to inject a liquid in the mixture cell where initially there is a known amount of other liquid. When the stirring is adequate, the mixture is homogeneous and the temperature everywhere in the binary mixture is the same. This is why “the mixture” can be represented as a single domain. It is in the energetic balance where all the additional terms corresponding to the thermal effects analyzed in this article must be incorporated.

The first of the effects, related to the heating or cooling of the injected liquid, can be evaluated with the expression *ρc_p_ f*Δ*T_mix_*, where *ρc_p_* is the volumetric heat capacity of the injected liquid, *f* is the injection flow and Δ*T_mix_*=*T*_0_–*T_mix_* is the difference of temperatures between the thermostat and the mixture. According to the information mentioned before, the domain equation would have the form:

(2) $$W_{\mathrm{mix}}+\rho c_{p}f\Delta T_{\mathrm{mix}}=C_{\mathrm{mix}}\frac{dT_{\mathrm{mix}}}{dt}+\sum_{1}^{N}P_{\mathrm{mix}\;k}(T_{\mathrm{mix}}-T_{k})+P_{\mathrm{mix}}(T_{\mathrm{mix}}-T_{0})$$

During the injection period, the heat capacity of the mixture *C_mix_* rises, producing an increase of the main time constant [15,19]. The thermal coupling between the domain that represents the liquid mixture and the neighbouring domains also change during the injection. Its most significant effect can be represented by the $P_{\mathrm{mix}}^{\prime }\Delta T_{0}$ term, where $P_{\mathrm{mix}}^{\prime }$ is the thermal conductivity of the coupling between the liquid mixture and the thermostat (through the stirring axis) and $\Delta T_{0}=T_{0}-T_{0}^{\prime }$ is the difference between the temperatures of the thermostat and the stirrer points. When there is a rise of the liquid volume in the cell, $P_{\mathrm{mix}}^{\prime }$ increases producing a disturbance in the baseline [20]. If a change in the density or viscosity of the mixture occurs, the power dissipated by the stirring process, *W_stirring_*, will also become modified. This fact will produce an additional change in the baseline. Adding these new terms to the equation of the energetic balance, we obtain:

(3) $$W_{\mathrm{mix}}+W_{\mathrm{stirring}}+\rho c_{p}f\Delta T_{\mathrm{mix}}-P_{\mathrm{mix}}^{\prime }\Delta T_{0}=C_{\mathrm{mix}}\frac{dT_{\mathrm{mix}}}{dt}+\sum_{1}^{N}P_{\mathrm{mix}\;k}(T_{\mathrm{mix}}-T_{k})+P_{\mathrm{mix}}(T_{\mathrm{mix}}-T_{0})$$

It is also convenient to indicate that, in an ITC, it is necessary to test experimentally that the stirring is adequate to assure the homogeneity of the mixture and that the determined mixture energy corresponds with the concentrations programmed in the experiment. If the liquids under study are very volatile, the stirring favours the evaporation and, consequently, it is precise to introduce an additional term associated with this effect [21]. Nevertheless, in the experiment shown below, this effect has been minimized. In future works, we will try to modify the Eq. 3 in order to include this effect. The thermal effects mentioned above superimpose in the calorimetric signal. In the following sections, these are identified and quantified in calorimetric experiments.

### 3.2. Effect due to the Difference between the Temperature of the Injected Liquid and the Temperature of the Mixture during the Mixing Process

**Figure 1.**
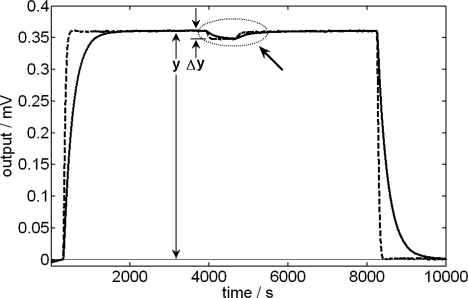
Calorimetric signal (continuous line) corresponding to a Joule dissipation. The arrow indicates the injection interval of 1.5 mL of *N*-methylformamide in 1 mL of the same liquid, with an injection flow of 2 μL/s. The discontinuous line shows the deconvolutioned signal. The injection effect is evaluated by the coefficient α*=*Δ*y/y.*

**Figure 2.**
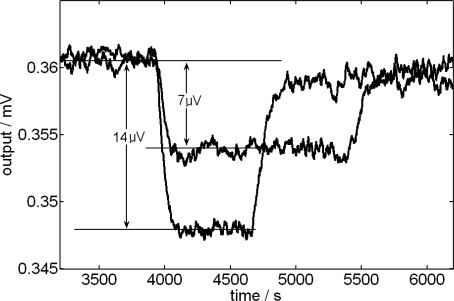
Injection effect during an electrical dissipation (see Figure 1). Extension of the deconvoluted signal in the injection interval for two different injection flows: 1 μL/s (Δ*y*=7 μV) and 2 μL/s (Δ*y* = 14 μV).

In the energetic balance of the domain which represents the content of the mixture cell (Eq. 3), it has been included the power due to the addition of liquid that is, initially, at the thermostat temperature *T*_0_. This power, *ρc_p_ f*(*T*_0_–*T_mix_*), always has opposite sign to the power developed in the mixture process because, if it is exothermic, *W_mix_* >0 and *T_mix_*>*T*_0_ and, if it is endothermic, *W_mix_* <0 and *T_mix_* >*T*_0_. In order to evaluate experimentally the effect produced by the injection of a determined liquid, a dissipation by Joule effect is caused and, once it has reached the stationary state, the liquid to be studied is injected on a known amount of the same liquid, producing in the calorimetric signal a decrease that is proportional to *ρc_p_ f*Δ*T*. Figure 1 shows the case of 1.5 mL of *N*-methylformamide on 1 mL of the same liquid for two injection velocities. In this case, the injection effect produces a signal reduction of 1.95% for an injection flow of 1 μL/s and of 3.9 % for a flow of 2 μL/s (Figure 2).

To be able to evaluate this effect, the calorimetric signal has been deconvolutioned by applying a derivative filter with a time constant τ*(t)* which varies linearly during the injection interval, from 230 to 260 s, and an integrating filter with τ^*^=40 s [19,22].

The relative reduction of the signal, α*=*Δ*y/y*, where y represents the output voltage, is determined experimentally for each liquid and for each injection flow used. Finally, the heat of mixing Δ*H* is calculated as follows:

(4) $$\Delta H=(1+\alpha )\frac{1}{K}\int_{t1}^{t2}y_{1}(t)dt$$

The curve *y*_1_(*t*) is the result of correcting the baseline to the deconvolutioned signal. *t*_1_ and *t*_2_ are the moments in which the signal *y*_1_ (*t*) is at the initial and final experimental zeroes of the calorimetric signal. *K* is the calorimetric sensitivity (in this case, *K*=391 mV/W [11]) obtained from chemical calibrations.

### 3.3. Effect due to the Increase of Liquid Volume in the Mixing Cell

The thermostat function is keeping constant the temperature and it serves as a reference for the measurement system and, that is the reason why the baseline should keep the same level. However, the experimental measures show that, after the injection has taken place, disturbances appear in the baseline. The disturbances are caused by two events: (1) the increase in the thermal conductivity between the content of the mixture cell and points of the stirring axis that are at a temperature $\left(T_{0}^{\prime }\right)$, slightly higher or lower than the thermostat temperature (*T*_0_). This effect is represented in Eq.3 by $P_{\mathrm{mix}}^{\prime }\Delta T_{0}=P_{\mathrm{mix}}^{\prime }(T_{0}-T_{0}^{\prime })$. (2) A second cause that alters the baseline is the variation of the power dissipated by the stirrer (*W_stirring_*) when the density and the viscosity of the liquid contained in the mixture cell have changed. Both effects need to be quantified for the correct determination of the heat of mixing.

**Figure 3.**
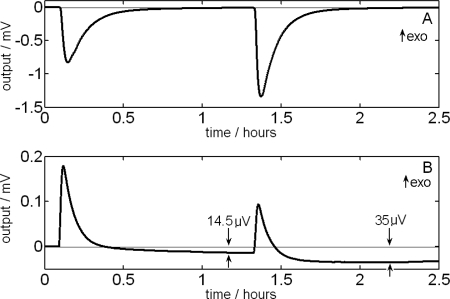
A) ITC-curve corresponding to two injection pulses of 50 μL of formamide into 1 mL of water. B) ITC-curve corresponding to two injection pulses of 50 μL of formamide into 1 mL of ethanediol. In both cases, the injection flow and the stirring velocity were of 1 μL/s and 60 rpm, respectively.

Figure 3A shows the calorimetric signal of an experiment corresponding to two injection pulses of 50 μL each (formamide is injected into 1 mL of water with an injection flow of 1 μL/s) using a constant stirring velocity of 60 rpm. The ITC-curve reveals a slight fall (−9 μV) in the baseline after the first injection. In this case, the relative variation of the baseline is not significant after the first injection, keeping this fall (−8.5 μV) after the second injection. In this case, the relative variation of the baseline is not significant on the signal as it is about 0.1% on the highest of the maximum of the first pulse. However, when the mixture energy is very low, it may occur that the relative variation of the baseline can be important, as in the case of the mixture formamide + ethanediol. In Figure 3B, it is shown the calorimetric signal that gives rise to two injection pulses of 50 μL each (formamide is injected into 1 mL of ethanediol with an injection flow 1 μL/s) using a stirring velocity of 60 rpm. The signal shows the fall of −14.5 μV in the baseline after the first injection and a fall of −35 μV after the second one. In this case, the jumps of the baseline are of 8% on the maximum of the first pulse and of 22% on the maximum of the second one. On the other hand, it is observed that the change in the baseline takes place during the injection. For this reason, it is more adequate to work with deconvolutioned curves because these are nearer the real process. In this case, the deconvolution process was carried out by using two derivative filters of time constants τ_1_=250 *s* and τ_2_=20 *s*. The voltage values were divided by the sensitivity to obtain the calorimetric signal in power units.

**Figure 4.**
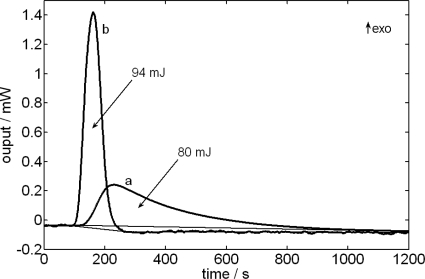
Energy of the second injection pulse corresponding to the Figure 3B (formamide + ethanediol). (a) Comparison among voltage values obtained from the ITC-curve and (b) from the deconvolutioned signal.

Figure 4 shows the case in which the relative variation of the baseline is higher, i.e., the second injection pulse of formamide on ethanediol (Figure 3B). The areas obtained after correcting the baseline are of 80 mJ for the case of the curve without filtering and of 94 mJ for the case of the filtered curve. The right result is the second one. The difference between both values reveals the need for doing a good correction of the baseline, in which it is always necessary to obtain an approximation of the dissipated power through a deconvolution of the calorimetric signal [19,22].

### 3.4. Effect due to the Stirring Velocity

**Figure 5.**
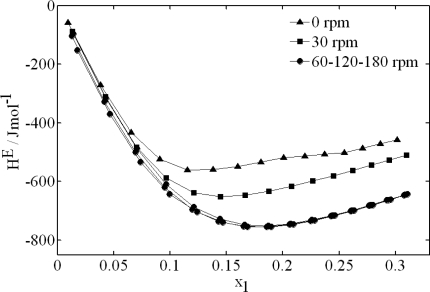
Excess molar enthalpies for ethanol (1) + water (2) mixtures obtained for different stirring velocities. It is shown the stretch obtained for ethanol injections on water.

A constant stirring velocity keeps the baseline at a constant level that will be able to be modified after the injection. The ideal stirring process is the one which assures the homogeneity of the mixture at every instant, not favouring the evaporation or increasing the signal noise. In order to analyze the effect that the stirring velocity has on the excess molar enthalpies, the ethanol + water system was analyzed. The measures consisted in 15 injection pulses of ethanol into 1 mL of water, each injection pulse during 100 s and a flow of 1 μL/s. Figure 5 shows final values of the excess enthalpy for different stirring velocities (0, 30, 60, 120 and 180 rpm).

For this binary system, it could be stated that, from 60 rpm, the stirring is sufficient. However, when the injection process is reversed (water is injected into 1 mL of ethanol), it is confirmed that when the stirring is increased up to 120 and 180 rpm, the absolute value of the mixture enthalpy decreases. This effect which has been also observed in other mixtures studied by us, it can be due to the fact that the evaporation has produced a reduction of the detected energy. For this reason, it is possible to conclude that, for exothermic liquid mixtures, the optimum stirring velocity is the one for which it is obtained the highest absolute value of the mixing enthalpy.

## 4. Conclusions

In this paper, the main thermal effects associated with the use of a calorimeter TAM2277-201/2250 by Thermometric (when this is used to measure heat of mixing of liquid mixtures) have been identified and quantified. Likewise, the equation of the energetic balance has been revised and those terms that represent the cited thermal effects have been incorporated.

It has been stated that the effect of the injection in the mixing cell produces a decreasing of the calculated energy is around 2% for injection flows of 1 μL/s, this percentage increasing when the injection flow and thermal capacity of the injected liquid, increases. It has been shown the need to correct the baseline through the deconvolution of the calorimetric signal when an important distortion is produced, in connection with the signal size. In the case considered in this work, it has been shown that a baseline which has not been corrected adequately produces an error in the determination of the mixture energy higher 15%. Finally, the effect of the change in the stirring velocity on the baseline and the measurement of mixture enthalpies, was determined, taking into account the need for assuring the homogeneity of the mixture, since, on the contrary, the calculated energies may lead to unreliable results.
